# Public Health Round-up

**DOI:** 10.2471/BLT.18.011118

**Published:** 2018-11-01

**Tackling air pollution, tackling noncommunicable diseases**

Air pollution air pollution is estimated to cause 7 million deaths per year, including 5.6 million deaths from noncommunicable diseases. This picture shows teenagers burning E-waste, imported from Europe, at a dump in Accra, Ghana. Waste burning is one of the causes of outdoor air pollution. (WHO/ Abraham Mwaura)

**Figure:**
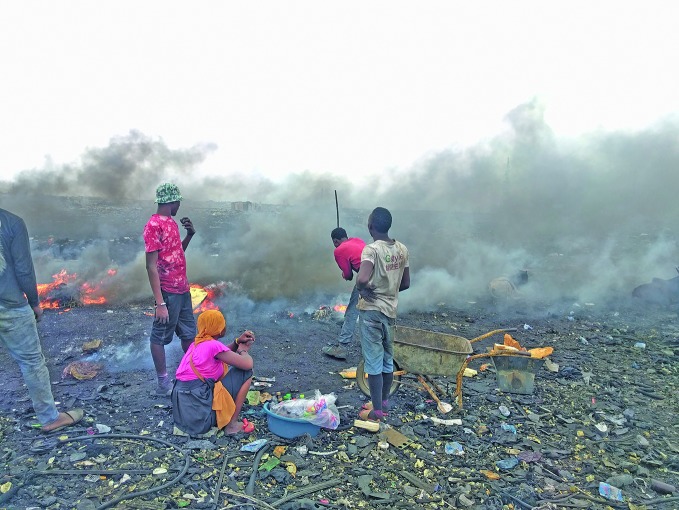
WHO/A. Mwaura

## Indonesia emergency 

The World Health Organization (WHO) is working with the Ministry of Health and other partners in Indonesia to provide support following the earthquake and tsunami that occurred in September.

WHO specialists have been helping to restore surveillance systems to ensure early detection of outbreak-prone diseases.

WHO is also supporting primary health organizations to ensure continuation of the national tuberculosis programme, including monitoring of drug-resistant tuberculosis patients and conducting inventory assessments for all districts in Central Sulawesi.

Immunization experts from WHO have been conducting cold chain assessments in three districts. Other specialists are coordinating and supporting Ministry of Health surveillance on water, sanitation and hygiene assessment and WHO is also advising on the provision of mental health and psychosocial support.

On 28 September, a 7.7 magnitude earthquake hit Central Sulawesi Province, affecting mainly Donggala district, Parigi Mountong district and Palu city. Fifteen minutes later, a tsunami hit Palu City.

As of 12 October, 2073 people were reported dead, 2549 were severely injured and 87 775 were displaced.

Some 20 health-care facilities have been affected, including one hospital.

http://bit.ly/2yhCQNx

## Vaccines prequalified

WHO prequalified a seventh inactivated polio vaccine (IPV), ShanIPV™ on 1 October.

WHO prequalification means that these vaccines are of acceptable quality, safe and efficacy, and thus recommended for bulk purchase by United Nations agencies and Gavi, the Global Vaccine Alliance, for eligible low and middle-income countries.

Since it is not a live virus, IPV carries no risk of vaccine-produced infection, unlike oral polio vaccine, which can give rise to vaccine-derived polio.

The use of IPV is recommended by the WHO Strategic Advisory Group of Experts (SAGE) for vaccines as part of the effort to eradicate all forms of polio.

Countries that have reached full immunization coverage with the oral vaccine are therefore encouraged to transition to IVP.

On 25 September, WHO prequalified Rotasil, an oral rotavirus vaccine, produced by Serum Institute of India. Rotasil is the first rotavirus vaccine that is stable in higher temperatures, making it suitable for use in low-income countries, where infrastructure and electricity supplies make refrigeration difficult.

Rotavirus is responsible for about 37% of deaths from diarrhoea among children younger than 5 years of age worldwide. WHO recommends that rotavirus vaccines be included in all national immunization programmes.

bit.ly/2CFf69N

## Air pollution conference

Government ministers, mayors, health and environment professionals and representatives of intergovernmental agencies gathered at the first Global Conference on Air Pollution and Health at the World Health Organization (WHO) headquarters in Geneva.

The event, from 30 October to 1 November, was organized by WHO in collaboration with United Nations Environment Programme, the World Meteorological Organization, the Climate and Clean Air Coalition World Bank, the United Nations Economic Commission for Europe, and the United Nations Framework Convention on Climate Change.

On the first two days of the conference, experts presented evidence, reviewed global progress and identified gaps and solutions. On the third day, participants aimed to develop a global goal and set of recommendations for an action agenda to reduce deaths due to air pollution as a contribution to achieving the sustainable development goals and the Paris Agreement.

Ambient air pollution in most cities exceeds WHO-recommended levels, in some cities by a factor of 10 or more, while household air pollution is a leading killer in low- and middle-income countries.

Government officials, urban mayors and representatives from civil society were invited to make commitments to the global advocacy campaign, Breathe Life, to meet WHO's Air Quality Guidelines and to reduce climate emissions.

bit.ly/2yldEGb

## WHO investment case 

WHO Director General, Tedros Adhanom Ghebreyesus, presented the Organization’s first-ever investment case at WHO headquarters on 19 September.

Based on WHO’s new five-year General Programme of Work, the case argues for US$ 14.1 billion over the next five years, or about US$ 2.8 billion per year, 14% above the WHO’s current base budget over a 5-year period.

The US$ 14.1 billion estimate includes a US$ 10 billion base budget, US$ 2.5 billion for humanitarian response and US$ 1.6 billion for polio eradication. The 14% increase refers to the increase in the base budget only, not the overall budget.

The investment case also stressed the importance of changing the way WHO is financed. Currently, more than 70% of the Organization’s budget is earmarked, leaving WHO with discretion on less than 30% of its financing.

This creates silos and internal competition for funds that makes it more difficult to deliver results.

The Director-General asked Member States and other donors to increase the proportion of discretionary funding.

“We need all countries to commit to flexible funding,” he said.

bit.ly/2RHcjBo

## Unnecessary caesareans

WHO issued new guidance on 11 October on non-clinical interventions designed to reduce unnecessary caesarean sections, the estimated incidence of which increased from 12% to 21% of all births globally between 2000 and 2015.

The new guideline, entitled *WHO recommendations on non-clinical interventions to reduce unnecessary caesarean sections*, recommends that health professionals use evidence-based clinical guidelines and regular audits of caesarean section practices in health facilities, and introduce financing arrangements that remove the incentive for over-prescription of caesarean sections.

The guidelines also recommend the provision of educational interventions to inform a meaningful dialogue with women and help women make informed decisions about mode of birth. These include counselling for women feeling excessive anxiety about giving birth.

A caesarean section can save the life of a woman and her baby. However, the procedure entails risks and should only be used when absolutely necessary.

bit.ly/2CGXgDJ

**Cover photo**

Residents carry their goods salvaged from the rubble at the Balaroa National Park, West Palu, Central Sulawesi, Indonesia after the earthquake and tsunami that struck on September 28.

**Figure:**
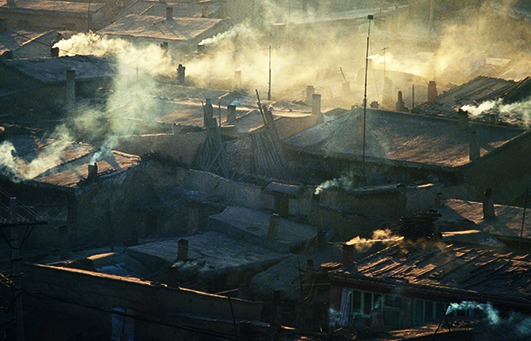
©UNICEF/Tirto.id/@Arimacswilander

## Children with cancer

WHO launched the Global Initiative for Childhood Cancer on 28 September, aiming to reach 60% survival for children with cancer by 2030, thereby saving an additional one million lives annually.

The initiative seeks to increase prioritization of childhood cancer through awareness–raising at global and national levels and to expand the capacity of countries to deliver best practice in childhood cancer care.

WHO will support governments to assess current capacities in cancer diagnosis and treatment including the availability of medicines and technologies; to set and cost priority cancer diagnosis and treatment programmes; and to integrate childhood cancer into national strategies, health benefits packages and social insurance schemes. 

http://bit.ly/2PvMItF

## UN high-level meeting on tuberculosis

World leaders pledged to ensure that 40 million people with tuberculosis receive the care they need by end –2022 on 26 September at the United Nations General Assembly in New York.

Governments also agreed to provide 30 million people with preventive treatment to protect them from developing tuberculosis.

Heads of state and government representatives attending this first-ever United Nations high-level meeting on tuberculosis agreed to mobilize US$ 13 billion a year by 2022 to implement tuberculosis prevention and care, and US$ 2 billion for tuberculosis research.

They also committed to take action against drug-resistant forms of the bacteria and prioritize human rights issues, such as stigma surrounding the disease.

The political declaration is the culmination of recent leadership commitments at global and regional level – including the 2017 Moscow Declaration to End Tuberculosis – to drive universal access, sufficient and sustainable financing, intensified research and innovation, and accountability across all sectors.

bit.ly/2IQrjJh

## Adolescents and alcohol

Adolescents in WHO’s European Region are consuming less alcohol than 12 years ago, according to a WHO report released on 26 September.

Despite the reductions, however, levels of consumption remain dangerously high and continue to be a major public health concern, according to *Adolescent alcohol-related behaviours: trends and inequalities in the WHO European Region, 2002–2014*.

Overall reductions in harmful drinking were the greatest in countries that traditionally have had higher prevalence, such as the United Kingdom of Northern Ireland and Great Britain and Scandinavian countries.

On 28 September WHO launched an alcohol control initiative to support a global target of reducing harmful use of alcohol by 10% by 2025.

http://bit.ly/2ydZOoK

**Looking ahead:**

1 December – World AIDS Day marks its 30th anniversary with the theme “Know your status”.

28 January – 5 February - 144th session of the Executive Board WHO headquarters.

29 January – 3 February - The Prince Mahidol Award Conference, Bangkok, Thailand. Theme: The Political Economy of NCDs: A Whole of Society Approach.

